# Impact of milk-to-vegetable fat ratio on infant formula emulsions stability

**DOI:** 10.1016/j.crfs.2026.101399

**Published:** 2026-04-09

**Authors:** Regis Badin, Claire Gaiani, Jeremy Petit, Jennifer Burgain, Laura Gailly, Preben Bødstrup Rasmussen, Colin Ray, Thorbjørn Vincent Sønderby, Søren Bang Nielsen, Florentin Michaux

**Affiliations:** a Université de Lorraine, Laboratoire D’Ingénierie des Biomolécules (LIBio), Nancy, 54000, France; b ARLA Foods Ingredients Group P/S, Videbæk, 6920, Denmark; c IUF (Institut Universitaire de France), Paris, France

**Keywords:** Infant formula, Emulsion, Stability, Creaming, Static multiple light scattering

## Abstract

Infant formula produced from specific ingredient fractions have attracted increasing interest in recent years. However, the removal of milk fat, combined with the lower emulsifying capacity of serum protein fractions from milk fractionation (e.g., Serum Protein Concentrate vs. Whey Protein Concentrate), increases the susceptibility of these formulations to destabilization, particularly when milk fat is substituted with vegetable oils. The stability of dairy ingredients in powdered form is directly linked to the stability of the emulsion prior to drying, meaning that any weakening of the interfacial layer or increased tendency toward droplet coalescence can significantly impact the physical quality and functionality of the final powder. To assess the effects of milk fat replacement on emulsion stability, different milk/vegetable fat ratios emulsions were produced (0; 0.06; 0.31; and 0.63). It was highlighted that emulsions containing the highest ratio of milk fat showed the narrowest droplet size distribution, with a significant decrease of the droplet mean diameter (d_50_), suggesting improved stability. In addition, emulsion destabilization was studied using Static Multiple Light Scattering, and higher milk fat content was linked to a significantly slower creaming rate. Moreover, droplet growth associated with coalescence and flocculation was also more limited for formulations presenting the highest milk fat content. These findings indicate that both milk fat substitution and the choice of milk protein fraction play a major role in emulsion stability. This study provides key insights for the design of novel milk-derived products.

## Highlights

• IF emulsion stability is strongly dependent on the milk fat/vegetable oil ratio.
• Formulations containing milk fat allow better stability to creaming.
• High Milk/Vegetable fat ratio slows droplet growth via coalescence and flocculation.
• Static Multiple Light Scattering is a relevant tool for the early identification of emulsion stability.

## Graphical abstract

**Figure:**
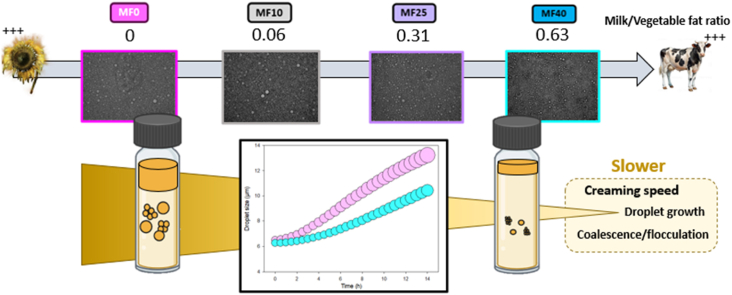

## 1 Introduction

Human breast milk is the gold standard for infant nutrition. However, breastfeeding is not always possible, whether due to medical, physiological, or practical reasons, making high-quality infant formula (IF) an essential alternative. Infant Formulas (IF), whose composition has been continuously adapted over the decades to meet the nutritional needs of infants, provide a safe and nutritious alternative (Blanchard et al., 2024; Kondrashina et al., 2024). However, the design of IF can be quite complex, as they contain numerous macro- and micro-components to meet infant development and growth. IF are thus processed food items with typical formulation of milk proteins, lactose or other carbohydrates, such as galacto- or fructo-oligosaccharides, vegetable oils or milk fat, micronutrients, and some other additives such as vitamins (Bakshi et al., 2023).

Most IF are based on bovine milk, as it is a highly nutritious and readily available raw material. However, its composition significantly differs from human breast milk, and adjustments are necessary to meet the dietary requirements of infants. Therefore, bovine milk–based formulas are often enriched with vegetable fats, minerals, and vitamins (Martin et al., 2016). Fat contributes roughly 50 % of the energy in infant formula and provides essential functional fatty acids (Koletzko et al., 2005). Among these, an optimal n-6/n-3 ratio of 5–15:1 is recommended to support the synthesis of long-chain polyunsaturated fatty acids, such as docosahexaenoic acid (DHA) and eicosapentaenoic acid (EPA), which are critical for infant neurodevelopment. The enrichment of formulas with vegetable oils, which are typically higher in n-6 fatty acids, may shift this ratio towards the upper end of the recommended range, potentially affecting the balance of polyunsaturated fatty acids available for neurodevelopment (Blanchard et al., 2024; Prosser et al., 2010). Because bovine milk is more costly than many plant-based alternatives (Delplanque et al., 2015), industrials are increasingly seeking to replace part of the milk fat with vegetable oils. The most commonly used vegetable fat for industrial IF include sunflower oil, coconut oil, soybean oil, rapeseed oil, among others. (Hageman et al., 2019; Mendonça et al., 2017). Up to now, milk fat has only been partially substituted by vegetal fats in commercial infant formulas. With the recent development of infant formulas, some products now combine fractions of vegetable oils with animal milk fat, while still meeting infants’ nutritional requirements by maintaining an appropriate n-6/n-3 fatty acid ratio, which supports optimal neural development (Delplanque et al., 2018). In cases where the milk fat is replaced by vegetal fat, the dispersed phase is composed of a vegetable blend that includes sunflower, coconut, soybean, or rapeseed oil, but can also be enriched in specific n-6/n-3 lipids (i.e., DHA/EPA). The main emulsifying agents used are soy lecithin or milk proteins and peptides. On the other hand, carbohydrates composition is quite similar across all IF formulation, as it systematically contains lactose and galacto- or fructo-oligosaccharides (Chen and Sun, 2021).

However, IF emulsions entirely composed of vegetable oils may undergo rapid destabilization, largely due to the absence of milk fat. This effect is attributed to Milk Fat Globule Membranes (MFGM) which contain polar lipids and specific membrane proteins that provides high emulsifying potential (Nie et al., 2024; Singh, 2019). Recent works have highlighted the critical role of MFGM. For instance, Li et al. (2024) showed that incorporating MFGM into model IF emulsions made with vegetable oils reduced fat droplet size and decreased zeta potential, while, Iung et al. (2025) proved that MFGM – whey protein interaction (i.e., β-lactoglobulin), improve emulsion rheological properties. Such emulsions made with MFGM also showed distinct rheological properties with higher apparent viscosity, and improved emulsion stability. On the other hand, He et al., 2017 went further and investigated which specific membrane proteins or lipids in MFGM are the main responsible for the stabilization role. Thanks to the study of various physicochemical parameters, such as fat droplet size distribution and emulsion stability, it was shown that smaller droplet size and better storage stability were obtained for emulsions containing milk fat globule membrane proteins, whereas no significant effects were found for emulsion enriched in the lipidic fraction. Indeed, MFGM are rich in phospholipids (15 - 30 % of total lipids), mainly phosphatidylcholine and phosphatidylethanolamine. This meets the study of Phan et al. (2016), suggesting that emulsions primarily stabilized with phospholipids may experience faster phase separation of the continuous and dispersed phases compared to protein-stabilized emulsions. When it comes to study emulsion destabilization phenomenon, Static Multiple Light Scattering (SMLS) technique is a powerful tool, as it allows to overcome common issues encountered with other techniques. Dynamic Light Scattering (DLS) can measure droplet size and zeta potential, offering information on emulsion physicochemical properties, but it cannot directly assess emulsion destabilization (Shi et al., 2024). SMLS techniques are based on the use of an illumination source (i.e., light or laser) with backscattering and/or transmission signals analyzed to study destabilization phenomena of emulsions. Thus, it can help to precisely monitor creaming or sedimentation kinetics at the top and bottom of the tube, respectively, or droplet size growth following coalescence, flocculation, or Ostwald ripening (Deb et al., 2022). For example, this technique was used by Bendjaballah et al. (2010) to optimize simple model oil-in-water emulsions, and to provide insights regarding the prediction of emulsion stability behavior depending on the emulsification process or temperature. The use of SMLS is also better than simple light scattering, as it allows to work at higher dispersed phase volume fraction, which is the case of IF emulsions (Shi et al., 2024). Predicting the behavior of IF emulsions prior to drying by SMLS is yet poorly described in the literature. Only Liu et al. (2024) investigated the effects of emulsification processes (i.e.; homogenization pressures and sterilization temperatures) on phospholipids composition and the structure of milk fat globule membranes. They proved that these processes strongly affect phospholipids composition and milk fat globule membrane structure, but also emulsion stability. They were thus able to link creaming and coalescence or flocculation over emulsion storage with the heat treatment applied.

Although previous studies have highlighted the relevance of MFGM in IF emulsions, the impact of varying milk-to-vegetable fat ratios on droplet size and emulsion stability remains poorly understood. This present study aims to characterize the physicochemical properties of model IF emulsions made with different milk/vegetable fat ratio formulated with SPC, with further evaluation of stability with SMLS.

## 2 Material and methods

### 2.1 Chemical composition and emulsification process of infant formula

Four different IF emulsions with various compositions were produced (Table 1). First of all, liquid samples such as Serum Protein Concentrate (SPC); Milk Protein Isolate (MPI), and Lactose were produced by membrane filtration of milk and provided by ARLA Foods Ingredients Group P/S, Videbæk, Denmark. Galacto-oligosaccharide (GOS) syrup, and Lecithin (Lecico-F600) were also provided (ARLA Foods Ingredients Group P/S, Videbæk, Denmark). Finally, Issigny cream (milk fat, 40% wt. lipids) and sunflower oil were purchased commercially.

**Table 1:** Chemical composition of investigated IF emulsions (MF0, MF10, MF25, and MF40).

Composition (g/200 g)	MF0	MF10	MF25	MF40
Aqueous Phase (%)	92.30	92.88	92.61	92.76
*SPC*	*83.38*	*83.38*	*78.88*	*76.50*
*MPI*	*33.41*	*33.41*	*32.09*	*30.80*
*Lactose*	*63.30*	*63.30*	*64.74*	*65.71*
*GOS Syrup*	*4.51*	*4.51*	*4.39*	*4.35*
*Cream (40% milk fat)*	*0*	*1.92*	*8.56*	*13.60*
*%PL from cream*	*0*	*0.03*	*0.15*	*0.23*

Dispersed Phase (%)	7.70	7.12	7.39	7.24
*Sunflower oil*	*15.04*	*13.48*	*10.99*	*8.68*
*Lecithin*	*0.37*	*0.37*	*0.36*	*0.36*

Milk/Vegetable fat ratio	0	0.06	0.31	0.63

Formulations (MF0, MF10, MF25, and MF40), representative of industrial infant formula compositions, were designed to reach target milk-to-vegetable fat ratios of 0, 0.06, 0.31, and 0.63, respectively. These ratios were selected based on industrial formulations developed by our partner Arla Foods Ingredients P/S (Videbaek, Denmark), ensuring technologically and nutritionally relevant substitution levels. The required amounts of cream were calculated assuming 40% fat in cream, while maintaining a final mass of 200 g for each formulation. For instance, MF25 corresponds to a formulation in which the amount of cream provides 25 % of the total lipid content, yielding a theoretical milk/vegetable fat ratio of 0.31 (Table 1). All of the ingredients were weighed as described in Table 1 to produce lab scale emulsion of 200 g. The aqueous phase was prepared by mixing SPC, MPI and Lactose mixed together with a T-25 Ultra Turrax equipped with a S25N-18G dispersed tool (IKA-Werke GmbH, Staufen, Germany) and at a temperature of 60 °C, which was controlled thanks to a Julabo Corio CP 200 F thermostated bath (Julabo GmbH, Seelbach, Germany). A few droplets of the dispersed phase (i.e., sunflower oil and lecithin) were added to the mixture as an anti-foaming agent. The mix was homogenized at 18000 rpm for 15 min. After that, cream was added and the aqueous phase was homogenized for 15 min at 18000 rpm again. Finally, the dispersed phase, composed of sunflower oil and lecithin was added dropwise and a final homogenization step of 10 min, still at 18000 rpm, was performed.

### 2.2 Droplet size characterization by laser granulometry and morphological analyses

Droplet size distribution was analyzed using a Mastersizer 3000 with a Hydro MV wet unit (Malvern Instruments, UK) supplied with two light sources at two distinct wavelength of respectively 632.8 nm (He-NE red light) and 470 nm (blue light) (Amutova et al., 2024). Emulsion droplet size was analyzed by laser granulometry just after the end of the emulsification process. Stirring rate of the dispersion unit was at least set at 1000 rpm and emulsion was added to reach a minimum of about 6 % of laser obscuration. Droplet size distributions were measured at least in triplicates for a measurement duration of 10 s. The emulsion droplet size (d_50_ and d_4,3_ μm) was determined from the volume distribution profiles, corresponding to the particle size at which the cumulative distribution reaches 50% (Li et al., 2023).

Concurrently, the droplet morphology was also studied with a Dinolite AM73915MZT numeric microscope (Dinolite, Ludres, France) at ambient temperature. The obtained micrographs were processed using the ImageJ software (NIH, MD, USA).

### 2.3 Surface tension measurements

Surface tension measurements have been performed on a K100 tensiometer (Krüss, Germany) using a Wilhelmy plate at 25 °C. The samples were thermostated for at least 10 min before the measurement began. Surface tension γ expressed in mN.m^−1^ has been recorded upon time until stabilization was achieved. γ = f (time) curves have been fitted using an exponential model to obtain g_f_ which corresponds to the surface tension value at the equilibriumEquation (1).

(1) $$\gamma =\gamma_{0}*\exp\left(\frac{t}{t_{1}}\right)+\gamma_{f}$$

Where γ is the surface tension at a given time t, γ_f_ is the equilibrium surface tension and g_0_ and t_1_ are constants related to respectively the value of γ at time 0, and the decrease of the exponential decline. Measurements have been performed in triplicate.

### 2.4 Emulsion stability

#### 2.4.1 Monitoring emulsion stability by SMLS

Emulsion stability was quantified by SMLS measurements with a Turbiscan LAB (MicroTrac Formulaction SAS, Toulouse, France). Briefly, this technique is based on the use of a pulsed near infrared light source (850 nm). Detection of emulsion destabilization (e.g.; creaming or sedimentation) was carried out by backscattered signal (BS) detection. In this case, 20 ml of the freshly prepared emulsion was poured into the sample cell and measurements were performed over 14 h by measuring the backscatter signal through the whole sample cell height every 30 min, in triplicates at 25 °C ± 2 °C. Creaming layer thickness was then determined over time and Turbiscan Stability Index (TSI), which is the cumulative sum of the difference between two scans, was calculated in the middle area (16-26 mm) of the tube thanks to Equation (2) (Perrin et al., 2023).

(2) $$TSI=\frac{1}{N_{h}}\sum_{t_{i}=0}^{t_{\max}}\;\sum_{h_{i}=h_{\min}}^{h_{\max}}\;\left|BS\;\left(t_{i},h_{i}\right)-BS\;\left(t_{i-1},h_{i}\right)\right|$$

Where $t_{\max}$ (h) is the measurement point at the time at which the TSI is calculated; h the sample height (mm); $h_{\min}$ and $h_{\max}$ the lower and upper height limits (mm) respectively, and $N_{h}=\frac{Z_{\max}-Z_{\min}}{\Delta h}$ the number of height positions in the selected zone of the scan and BS the considered backscattered signal (BS in this case, as T < 0.2%).

#### 2.4.2 Mathematical modelling of creaming rate, TSI, and droplet size growth

##### 2.4.2.1 Creaming

In order to identify differences in the destabilization kinetics mechanisms that may come from the type of fat, the creaming kinetic was modelled using the Hill equation, which is described in Equation (3) (Fournaise et al., 2021).

(3) H(t)=h0+(h∞−h0)×tnt12n+tn

Where $h_{0}$ is the initial cream thickness at the top of the tube (mm); $h_{\infty }$ the final cream thickness (mm); t12 the time required to reach the half value of $h_{\infty }$; and n the Hill model constant.

Finally, Creaming Rate (C_R_) was calculated as the slope at t12 and was determined as followed Equation (4):

(4) CR=n(h∞−h0)4t12

##### 2.4.2.2 Temporal evolution of TSI

The TSI evolution over time in the middle of the tube (16 to 26 mm) was modelled using the logistic model, as described in Equation (5). It allows to describe the instability evolution of the different emulsions. This model is similar to the Hill model but allows to work with slope time delay.

(5) H(t)=h0+(h∞−h0)1+e−(t−t12)T

Where $h_{0}$ is the initial TSI value; $h_{\infty }$ the final TSI value; t12 the time to reach the half value of $h_{\infty }$; and T the characteristic time.

Finally, the TSI rate (${TSI}_{S}$) was calculated as the slope at t12 following Equation (6):

(6) $${TSI}_{S}=\frac{\left(h_{\infty }-{\;h}_{0}\right)}{4T}$$

##### 2.4.2.3 Droplet size growth

The droplet size growth (μm.h^−1^) was calculated over time in the middle of the tube (16 to 26 mm) for all emulsion thanks to Equation (7) (Turbisoft LAB 3.0.2.0, Microtrac, France). This equation is based on the Mie theory and allow to determine the mean size of scattering objects in the sample using the continuous phase refractive index (1.348) and the dispersed phase refractive index (1.456), which were determined using a DR-A1/NAR refractometer (Atago, Japan) at ambient temperature (25 °C).

(7) $$BS=\sqrt{\frac{3\phi \;\left(1-g\left(d\right)\right)Q_{e}\left(d\right)}{2d}}$$

Where BS is the backscatter signal, ϕ the dispersed phase volume fraction (% v/v); d the droplet size diameter (μm); and $Q_{e}$ and g are Mie theory optical parameters.

In this case, droplet size growth was modelled using the Hill model, as detailed in Equation (8).

(8) d(t)=d0+(d∞−d0)×tnt12n+tn

Where $d_{0}$ is the initial droplet diameter; $d_{\infty }$ the final droplet diameter; t12 the time to reach the half value of $d_{\infty }$; and n a model constant (−).

### 2.5 Statistical analyses

Statistical analyses were performed using the Sigmaplot software in the version 15.0 (Systat Software, Chicago, USA). Data were analyzed using a one-way ANOVA test with a Tukey-HSD means comparison on 3 replicates and at levels of significance for the following p values: non-significant for p>0.05 and significant for ∗p<0.05;∗∗p<0.01;∗∗∗p<0.001.

## 3 Results and discussion

### 3.1 Physical properties of IF emulsions

**Fig. 1:**
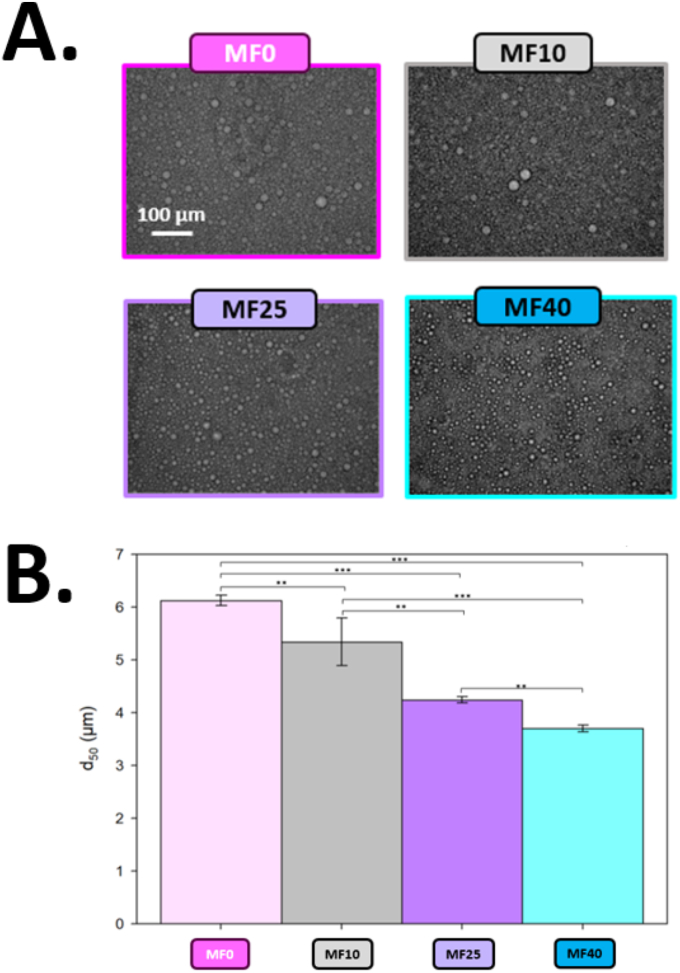
A. Numeric optical microscopy micrographs of four different emulsions, MF0; MF10; MF25; and MF40. B. Average droplet size (d_50_) of MF0; MF10; MF25; and MF40 determined by laser granulometry. (∗p<0.05;∗∗p<0.01;∗∗∗p<0.001).

Digital microscopic images were taken directly after the emulsification process. Images suggest that the polydispersity of the fat globules size is markedly different depending on the amount of vegetable fat (Fig. 1A). In MF0, where the IF contained only vegetable fat (as described in Table 1), the droplets were larger and showed a more heterogenous size distribution compared to formulations containing milk fat. As the milk fat content increased, the fat globules became smaller and more uniformly distributed, especially for MF25 and MF40.

To support microscopic observations, laser granulometry measurements were performed to measure the median fat droplet size (d_50_) of the different IF emulsions. The results obtained in Fig. 1B were consistent with those previously stated, as the emulsion containing vegetable fat only (MF0) showed the highest d_50_ value of 6.12 ± 0.09 μm. As the milk fat content increases in IF, the median droplet size decreases. Indeed, the d_50_ decreases from 4.98 ± 0.22 μm for MF10 (i.e.; 10 % of milk fat), to 4.24 ± 0.06 μm for MF25 (i.e.; 25 % of milk fat) and down to 3.70 ± 0.06 μm for the emulsion MF40 (i.e.; 40 % of milk fat), which had the highest milk/vegetable fat ratio.

It is important to note that the droplet size range of the IF obtained in this study might be higher than others already described in the literature. However, this observation might be due to the differences in the emulsification process used in this work. For example, Zhu et al. (2025) studied the impact of MFGM and egg yolk addition on lipid digestion of IF, produced emulsion with a monomodal distribution centered on the 400-500 nm range using high pressure homogenizer. In another study, Li et al. (2023) reported also similar sizes for similar models of IF emulsions, with a d_(4,3)_ of 5.5 ± 0.3 μm for an emulsion made from WPI.

Droplet size below the micron are usually obtained using high pressure homogenizer rather than dispersing tools (Paquin, 1999). Nevertheless, reaching a droplet size below the micrometer might be difficult even using high pressure homogenization, and droplet sizes ranging from 1 to 10 μm may still be observed (De Figueiredo Furtado et al., 2021; McCarthy et al., 2012). This technique generally enhances stability, since smaller droplets are less prone to destabilization during storage. However, high shear emulsification is still the most common way of making emulsion, and droplet size distribution still generally follows a log normal distribution despite the repetitive and random breakup of drops. This assessment was confirmed by L’Estimé et al. (2024), who showed that fat globule size after the emulsification process mostly relies on intrinsic parameters, such as rotation speed, mixer geometry, pressure, and continuous phase viscosity rather than the process itself.

As previously stated, and observed from Fig. 1B and from Supplementary Material (Table 1; Fig. 1) average fat droplet size (d_50_) is directly correlated to the milk-vegetable fat ratio. As milk fat content increases, particle size decreases, proving the key role of fat source (i.e., vegetal or animal) on the IF emulsion physical properties. Indeed, d_50_ decreased from 6.12 ± 0.09 μm for MF0 to 3.70 ± 0.06 μm for MF40. Similarly, the volume-weighted mean diameter (d_4_,_3_), which better reflects the contribution of larger droplets to the overall volume distribution, decreased from 24.26 ± 3.59 μm for MF0 to 8.05 ± 0.30 μm for MF40, confirming the presence of size differences between the different IF formulations. In addition, particle size distribution was more heterogeneous in samples with a low milk/vegetable fat ratio, as illustrated by the volume distribution profiles (Supplementary Material, Fig. S1). As stated by Murphy et al. (2020), such heterogeneity can arise from either poor emulsification efficiency or compositional differences, as is the case here with major changes in fat sources. Because droplet size is directly correlated with emulsion stability, especially from creaming and size variation phenomena (Kupikowska-Stobba et al., 2024), this observation suggests that the higher heterogeneity observed in our study may predispose these emulsions to faster destabilization phenomena (e.g., creaming, flocculation, or coalescence) during storage and reconstitution.

### 3.2 Destabilization phenomena of IF emulsions during storage

**Fig. 2:**
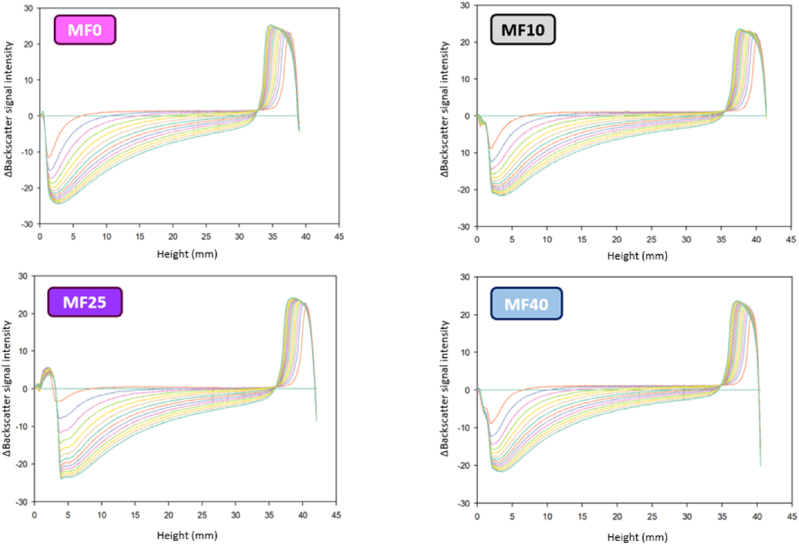
Following emulsion stability over time: SMLS profiles obtained for emulsion presenting increased level of milk fat (MF0; MF10; MF25; and MF40).

SMLS technique allows the precise identification of emulsion destabilization phenomena that occur (i.e.; creaming, sedimentation, coalescence and/or flocculation) (Goscianska et al., 2019). The destabilization kinetics of the different emulsions (i.e., MF0, MF10, MF25, and MF40) were obtained and analyzed over 14 h (Fig. 2).

No significant difference in terms of profile shape were noticed between samples. For all of them, a decrease of the backscattering signal over time can first be observed at the bottom of the emulsion (clarified layer). On the opposite, an increase of the backscattering signal can be seen at the top of the emulsion (creaming layer), at height values that varies around 30 to 40 mm. Some slight signal variation (i.e.; decrease of the backscattering signal) can also be detected in the middle area, ranging at heights between 16 and 26 mm. These variations in the backscattering signal indicate that the emulsions undergo identifiable destabilization phenomena during storage. As stated by Shi et al. (2024), the identification of signal variations at the top and in the middle of the tube (i.e.; increase of the backscattering signal) are respectively due to creaming and coalescence and/or flocculation phenomena. This observation is consistent with previous findings, as these destabilizations phenomena are the main ones affecting IF (Chen and Sun, 2021). In a recent study Liu et al. (2024) reported similar destabilization profiles and phase separation behaviors for IF as those observed in this study. As observed by Degrand et al. (2016) on conventional oil-in-water emulsions, a progressive increase of the backscattering signal at the top of the tube means that creaming occurs, as a fat layer forms at the surface. This observation is quite logical with this phenomenon, as it is a gravity-driven phenomenon that relies on density differences between the continuous and the dispersed phase (Debnath et al., 2015). Although IFs are destined to be dried into powder shortly after the emulsification process, it is of relevance to better understand the destabilization kinetics. Indeed, powder stability and powder reconstitution are highly dependent on the emulsion stability prior drying. For example, Rodríguez Arzuaga et al. (2022) observed the impact of pasteurization temperatures and total solids contents on IF stability and proved that flocculation and coalescence observed prior drying was linked to poorer powder stability after reconstitution, as higher particle size was observed with higher total solids contents and temperatures.

#### 3.2.1 Creaming kinetics over storage

**Fig. 3:**
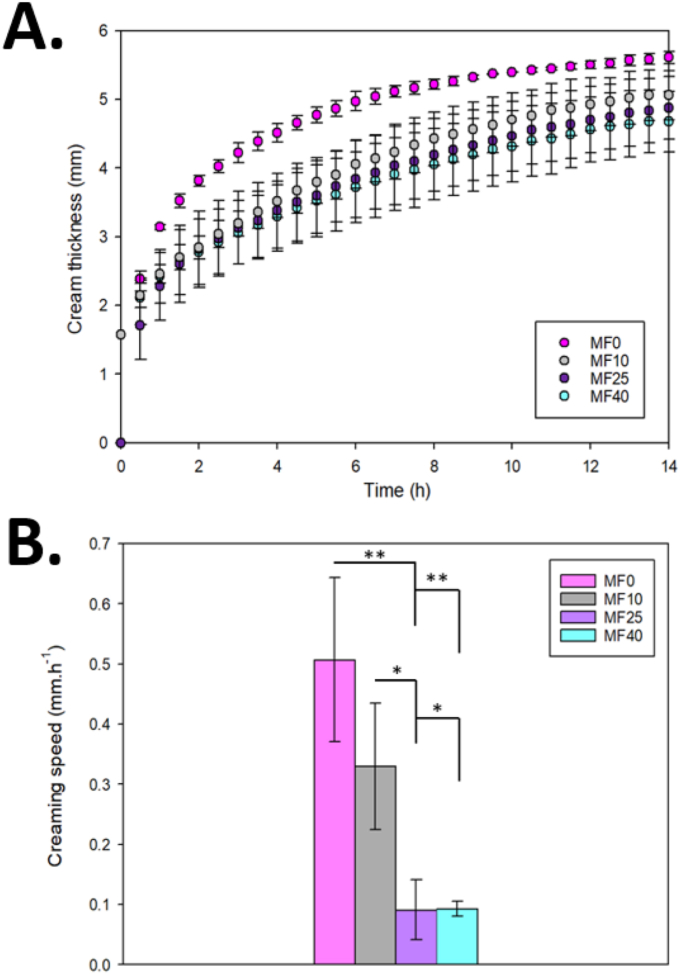
A. Creaming kinetics over storage of all different emulsion (MF0, MF10, MF25, and MF40). B. Associated creaming rate of all emulsions calculated by Hill model. (∗p<0.05;∗∗p<0.01;∗∗∗p<0.001).

As previously stated, creaming was observed for all emulsions, whatever the milk/vegetable fat ratio, that varies from 0 (MF0) to 0.63 (MF40) (Fig. 2). To further discriminates the differences in destabilization phenomena between studied samples, creaming kinetics of all IF emulsions was studied (Fig. 3). It is important to note that creaming mostly relies on Stokes’ law (Chanamai and McClements, 2000). Thus, creaming behavior is highly dependent on density differences between fat droplets and bulk solution, continuous phase viscosity (i.e.; polysaccharides content for the case of IF), but particularly on particle size (Degrand et al., 2016; Robins, 2000). Here, continuous phase viscosity was highly dependent on the GOS syrup addition during the emulsification process, and the same amount was added for all emulsions (Table 1). Thus, the differences of creaming kinetics observed for investigated emulsions were then mainly dependent on fat droplet size.

First, it was observed that final cream layer thickness at the top of the tube (after 14 h) was similar for all emulsions, with values ranging between 4 and 5 mm, as phase separation occurs (Fig. 3A). This result is consistent with the emulsion composition, as the dispersed phase percentage was similar for all emulsions, with changes only in the milk/vegetable fat ratio (Table 1). However, despite similar SMLS profiles, distinct creaming behaviors were observed. Thanks to Hill model Equation (3), the creaming rate was determined by using the slope at half height. Differences were observed between formulations (Fig. 3B), with the highest rate (0.51 ± 0.14 mm h^−1^) for MF0. In comparison, MF10 (containing only 10% of milk fat relative to the total lipid content), exhibited a lower creaming speed of 0.33 ± 0.11 mm.h-1. This value was not significantly different from MF0. However, a significant effect of milk fat addition was noticed starting from 25 % milk fat within total fat with markedly lower creaming rates of 0.09 ± 0.05 mm.h-1 and 0.09 ± 0.01 mm.h-1 for MF25 and MF40, respectively. Increasing the milk-to-vegetable fat ratio enhanced the emulsion's resistance to creaming, consistent with the smaller droplet size (d_50_) observed at higher ratios (Fig. 1B).

Particle size distribution and morphology (Fig. 1), permitted to highlight that larger and heterogenous fat globules were obtained at low milk-to-vegetable fat ratios. This suggests that such emulsions may be more prone to destabilization. The presence of larger fat globules immediately after emulsification could indicate that coalescence at best begins at the very onset of emulsion storage. Coalescence has indeed been reported as one of the main destabilization mechanisms in IF. For example, Rodríguez Arzuaga et al. (2022) stated that an increase of particle size in IF after reconstitution (using anionic surfactant, such as sodium dodecyl sulfate) mainly involved coalescence of dispersed fat globules. Additionally, (Chen and Sun, 2021) reported that the emulsification process also plays a key role on the destabilization phenomenon. Indeed, the production of IF always involves high shearing processes, such as dispersion and agitation tools or even high-pressure homogenization. They indicate that high temperature or high pressure induce fat droplet coalescence, which can further lead to impairment of IF powders, such as high surface free fat coverage, leading to poor wettability and thus poor reconstitution ability. It can even lead to increased susceptibility to caking and stickiness, or even fatty acids oxidation.

#### 3.2.2 Size variation of fat globules over storage

Beyond creaming, variations in fat globule size throughout the sample was also observed in the middle area of the tubes, as detailed in Section 3.2.1. and in Fig. 2, with a decrease of the backscattering signal observed. Thanks to SMLS, it is possible to study such size variations, which are mainly caused by coalescence, flocculation, or even Ostwald ripening (Degrand et al., 2016; Tehrani-Bagha et al., 2016). To minimize the influence of initial droplet size and creaming on TSI measurements, data were collected at the middle of the sample tube (16 to 26 mm of height on all samples), allowing the evaluation of coalescence and flocculation independently of creaming effects, which mostly relies on continuous phase viscosity and initial droplet size (Santos et al., 2022). The viscosity of the continuous phase was maintained across all formulations, and was mainly dependent on the addition of galacto-oligosaccharide syrup, which is the same between all formulations (Table 1).

**Fig. 4:**
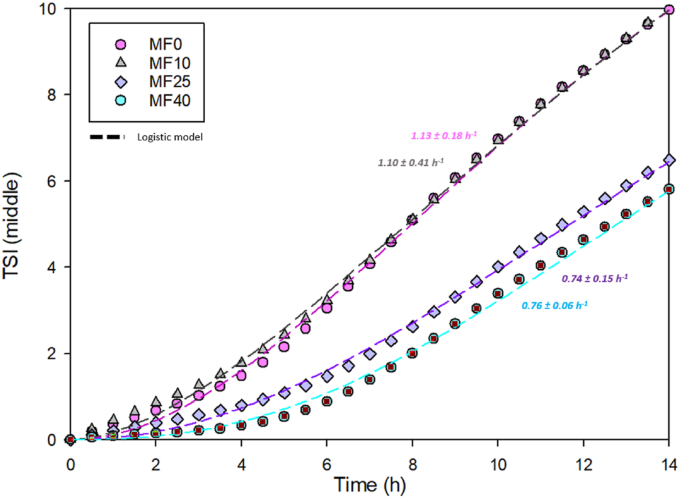
Turbiscan Stability Index (TSI) evolution in the middle area of all emulsions (MF0, MF10, MF25, and MF40). TSI evolution was calculated using the logistic model as described in Equation (4).

In order to first identify if there were significant differences between all emulsions, the Turbiscan Stability Index (TSI) of MF0, MF10, MF25, and MF40 was calculated (Fig. 4). For studying emulsion stability, TSI provides a rapid and effective means of predicting stability during storage through backscattering signals. In the case of IF, it proved particularly useful for assessing destabilization phenomena related to storage temperatures. By analyzing the height differences between consecutive scans across the entire emulsion range, Liao et al. (2024) proved that fat globule size initially increased with storage and for higher temperatures, due to enhanced Brownian motion that promoted particle agglomeration. They also showed that TSI values were highly dependent on storage time and temperatures, as higher TSI (i.e., greater destabilization) were noticed in the case of improper storage conditions, and were attributed to coalescence and/or flocculation in the middle of the sample.

In this work, creaming kinetics and TSI evolution over time both permitted to evidence a marked influence of milk/vegetable fat ratio on emulsion stability (Fig. 4). A threshold between 10 and 25 % milk fat over total fat was observed for the improvement of emulsion stability by slowing of coalescence and/or flocculation kinetics. Modelling of TSI curves permitted to prove that MF0 and MF10 shared the same TSI evolution rate (1.10 ± 0.41 and 1.13 ± 0.18 TSI. h^−1^, respectively) which markedly differ from the ones of MF25 and MF40 (0.74 ± 0.15 and 0.76 ± 0.06 respectively). It is worth noting that TSI temporal evolutions were almost identical for MF25 and MF40. The same observations were made from creaming results (Fig. 3). Indeed, a significant reduction of creaming rate upon milk fat addition was noticed from MF25. The same milk/vegetal fat ratio threshold (i.e.; 0.31/MF25) as for coalescence and flocculation was identified for the creaming mechanism. Initially, the molecular events leading to creaming — such as droplet coalescence and flocculation — occur throughout the sample. As creaming progresses, a gradient in the dispersed phase volume fraction develops, with higher droplet concentrations at the top. Larger fat globules are more prone to migrate upward in accordance with Stokes’ law (Robins, 2000), emphasizing that particle size variation in the middle area of the sample is critical to understand both coalescence/flocculation kinetics and the influence of the milk/vegetable fat ratio on emulsion stability. Consequently, studying droplet size evolution provides key insight into the mechanisms governing creaming and overall emulsion behavior.”

**Fig. 5:**
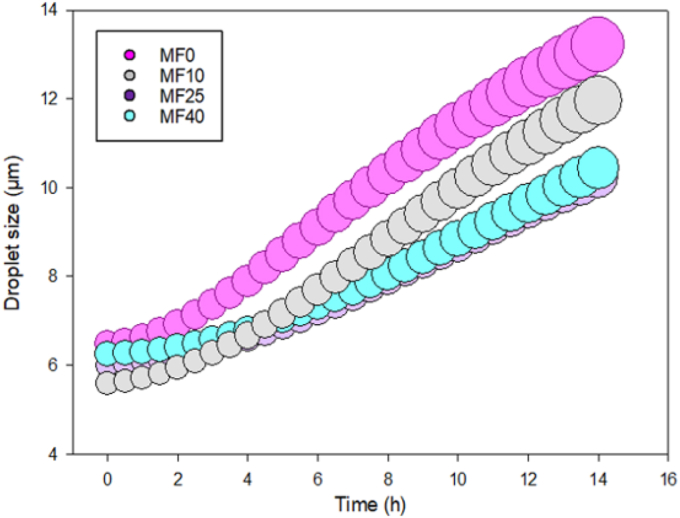
Coalesced/flocculated fat droplet size evolution over emulsion storage time for all emulsions (MF0, MF10, MF25, and MF40). The dot size also represents the calculated droplet size at a *t* time.

To study how the milk/vegetable fat ratio can influence coalescence and flocculation kinetics, the Mie theory was used to calculate the droplet size and growth over time Equation (7). Such models have already proved to be efficient for calculating droplet sizes over a large size range (generally from about 10 μm to 1 mm) (Garcia et al., 2022). In the same way that it was observed for the TSI evolution over time and creaming, two main trends were observed (Table 2) (Fig. 5). The model used in this work was not able to predict significant differences in initial (t_0_) and final (t_fin_) droplet sizes. Indeed, initial sizes and final sizes were calculated at 6.48 ± 0.82 and 13.21 ± 2.36 μm for MF0; 5.58 ± 0.72 and 11.96 ± 1.94 μm for MF25; 5.98 ± 0.54 and 10.23 ± 2.54 μm for MF25; 6.25 ± 0.15 and 10.42 ± 0.37 μm for MF40. This might be due to the fact that the model was used at the lowest range of its possible application (at around 10 μm) (Garcia et al., 2022). However, when it comes to study the droplet size growth kinetic, this model based on the Mie theory is still powerful. Despite this, the size variation, which is estimated around 5 μm after 14 h, still suggest that fat globules in the IF emulsion might be affected by coalescence and flocculation (see Table 3).

**Table 2:** Initial size (t_0_); Final size (t_fin_): and droplet size growth rate (mm.h^−1^) calculated thanks to Equation (7).

	MF0	MF10	MF25	MF40
Initial size (t_0_) (μm)	6.48^a^ ± 0.82	5.58^a^ ± 0.72	5.98^a^ ± 0.54	6.25^a^ ± 0.15
Final size (t_fin_) (μm)	13.21^a^ ± 2.36	11.96^a^ ± 1.94	10.23^a^ ± 2.54	10.42^a^ ± 0.37
Droplet size growth rate (μm.h^−1^)	0.50^a^ ± 0.13	0.47^a^ ± 0.16	0.31^a^ ± 0.12	0.31^b^ ± 0.01

**Table 3:** Surface tension and Zeta potential values obtained for MF0 and MF40 emulsions.

	Surface tension (mN.m^−1^)	Zeta potential (mV)
MF0	47.8 ± 0.2	−29.5 ± 0.2
MF40	39.6 ± 0.1	−28.8 ± 0.3

Modelled droplet size kinetics made appear differences in droplet size growth among samples. Indeed, Fig. 5 highlights a marked reduction of droplet growth rate for MF25 and MF40. However, a significant difference in droplet size growth was only observed between MF0 and MF40, with values calculated at 0.50 ± 0.13 and 0.31 ± 0.01 μm h^−1^ respectively. Thus, using a high milk/vegetable fat ratio (i.e.; MF25 and MF40) could limit coalescence and flocculation by reducing destabilization. Nevertheless, the effect of milk fat recovery on vegetable fat IF can still be attributed to the presence of higher milk fat globule membrane content, which are naturally present in milk and possess emulsifiers components, as described for creaming phenomenon in 3.2.2. (He et al., 2017; B. Li et al., 2024). Despite the ability of the model to describe slight size variation kinetics in the micron range, the distinction of coalescence and flocculation was impossible in this case. However, the micrographs presented in Fig. 1A suggest that coalescence, or maybe Ostwald ripening, might be the main destabilization phenomenon occurring over flocculation, as no flocculated clusters were observed right after the emulsification process, even for the less stable emulsions (i.e., MF0 and MF10) (Kawaguchi, 2016; Robins and Hibberd, 1998).

Table 2 shows the initial size (t_0_), final size (t_fin_) and droplet size growth rate for the different formulations. Although the SMLS model does not allow statistical comparisons between the initial and final droplet sizes, significant differences in droplet growth rate were observed between the two extremes, MF0 and MF40, reflecting the effect of increasing the milk-to-vegetable fat ratio (i.e., from 0 to 0.63). As it is also shown in Fig. 2 and Table 2, the rates of coalescence and flocculation are generally slower than the creaming kinetics. Increasing the milk-to-vegetable fat ratio in infant formula improved stability against all three phenomena. Interestingly, even when coalescence and flocculation rates exceed those of creaming, emulsion stability can still be enhanced. This is because larger droplet aggregates (i.e., flocs), formed at higher flocculation rates, can generate a weak particle network that resists gravitational forces, thereby slowing down the overall creaming process (Damodaran, 2006; Degrand et al., 2016).

#### 3.2.3 Links between IF emulsion stability and MFGM

All the observations also support the key role of the milk fat globule membrane present in the milk fat. Indeed, MFGM possess a tri lamellar structure that contains roughly 75 % of lipids and 25 % of proteins (Maldonado-Celis et al., 2019; Nie et al., 2024). Among its lipid components, 10 to 30 % are phospholipids (Pan et al., 2023). To investigate the interfacial composition of the emulsions and the role of MFGM in stabilization, surface tension of the aqueous phase and zeta potential of MF0 (less stable emulsion; no MFGM) and MF40 (most stable emulsion; highest MFGM content) were measured (Table 3).

The results showed similar zeta potential values for emulsions prepared with and without cream, suggesting that the presence of MFGM fragments did not significantly modify the overall electrostatic surface charge under the experimental conditions. This observation may be explained by the dominant contribution of adsorbed milk serum proteins at the interface, which likely governs the surface charge of the droplets, as observed by Silva et al. (2021) in oil–water emulsions prepared with varying levels of casein and whey proteins. This is also consistent with the formulation of the investigated systems, which contain relatively high levels of milk proteins (MPI and SPC), whereas the estimated amount of phospholipids introduced through cream addition remains comparatively low (0–0.23%), and therefore is unlikely to substantially alter the overall electrostatic characteristics of the droplet interface.

In the opposite, surface tension values were much lower (47.8 ± 0.2 and 39.6 ± 0.1 mN m^−1^ for MF0 and MF40 respectively) (Table 3) than that of pure water (72.5 mN m^−1^ at 25 °C), confirming the presence of surface-active molecules. The aqueous phase of MF40 exhibited a notably lower surface tension than MF0, suggesting that MFGM components from cream further reduced the surface tension. This effect likely extends to the oil–water interface, where MFGM-derived phospholipids and membrane proteins may decrease interfacial tension, facilitating droplet disruption during shearing and contributing to the smaller droplet sizes observed as cream content increases from MF0 to MF40 (Dickinson, 2010; Sparr and Wennerström, 2011).

Apart from its health benefits, such as a better digestibility, MFGM may act as natural emulsifiers thanks to its high content in polar lipids and proteins (Jukkola et al., 2019). As they are naturally present in the buttermilk fraction, the loss of these bioactive molecules might be the key for a better understanding of vegetable fat infant formula destabilization. Only a few works already describe the addition of isolated MFGM in IF emulsions. For example (Li et al., 2024), proved that the addition of MFGM in vegetable fat emulsions allowed improved physicochemical properties, such as a lower fat droplet size, improved rheological behaviors (i.e., increased viscosity), leading itself to better stability over storage and especially upon creaming. They even showed that MFGM helped not only to improve emulsion characteristics prior drying, but also IF powders bulk properties. He et al., 2017 investigated which components of phospholipids or proteins contribute to emulsion stabilization. They found that MFGM transmembrane proteins were the primary emulsifiers (at concentrations of 1–4%), whereas MFGM phospholipids alone were unable to stabilize the prepared emulsion. However, they confirmed that the addition of MFGM proteins led to smaller droplet sizes, and reduced shear stress suggesting that MFGM and its constituents may act as key stabilizers in the emulsion studied here particularly at higher milk-to-vegetable fat ratios. It is also noteworthy that milk fat recovery contributed to reducing the variability in creaming speed (Fig. 3B). Indeed, MF0 presented a high variability whereas emulsion with the lowest creaming speed (MF25 and MF40) showed less variability. This further supports the notion that optimizing the milk-to-vegetable fat ratio is critical for enhancing emulsion stability.

## 4 Conclusion

This study shows that the milk-to-vegetable fat ratio strongly influences the physical properties and stability of IF emulsions. Partially or completely replacing milk fat with vegetable fat profoundly influences key emulsion properties (most notably droplet size) which in turn directly affects its temporal stability, including susceptibility to creaming, coalescence, and flocculation. Higher vegetable fat content led to bigger droplets and reduced stability, while higher milk fat levels (MF25 and MF40) slowed creaming and droplet growth, as confirmed by SMLS and Mie theory modelling. These effects are largely attributed to the superior emulsifying capacity of the milk fat globule membrane. As the proportion of vegetable fat increases in IF, a deeper understanding of the functional role of the dispersed phase (and how its composition influences interfacial organization and droplet behavior) is essential for designing stable and robust formulations.

## Declaration of competing interest

The authors declare that they have no known competing financial interests or personal relationships that could have appeared to influence the work reported in this paper.

The authors declare the following financial interests/personal relationships which may be considered as potential competing interests:

Claire Gaiani reports financial support was provided by Arla Foods Ingredients. Florentin Michaux reports article publishing charges was provided by University of Lorraine. If there are other authors, they declare that they have no known competing financial interests or personal relationships that could have appeared to influence the work reported in this paper.
