# Supplementary material for: Impact of milk-to-vegetable fat ratio on infant formula emulsions stability
Source: Curr Res Food Sci. 2026 Apr 9;12:101399. doi: 10.1016/j.crfs.2026.101399 (PMC13092700; doi:10.1016/j.crfs.2026.101399)
Supplement: Multimedia component 1 [file mmc1.docx]

**Impact of Milk-to-Vegetable Fat Ratio on Infant Formula Emulsions Stability**

**Supplementary Material**

**Table 1:** Volume-weighted diameter (d₄,₃; µm) and median particle size (d_50_; µm) obtained from fat globule size distribution measurements.

|  | d_50_ (µm) | d_4,3_ (µm) |
| --- | --- | --- |
| MF0 | 6.12 ± 0.09 | 24.26 ± 3.58 |
| MF10 | 5.51 ± 0.23 | 24.94 ± 3.82 |
| MF25 | 4.32 ± 0.06 | 7.99 ± 0.30 |
| MF40 | 3.70 ± 0.06 | 8.05 ± 0.30 |

**Figure 1:** Volume distribution of different infant formula formulations (MF0; MF10; MF25; and MF40)

**
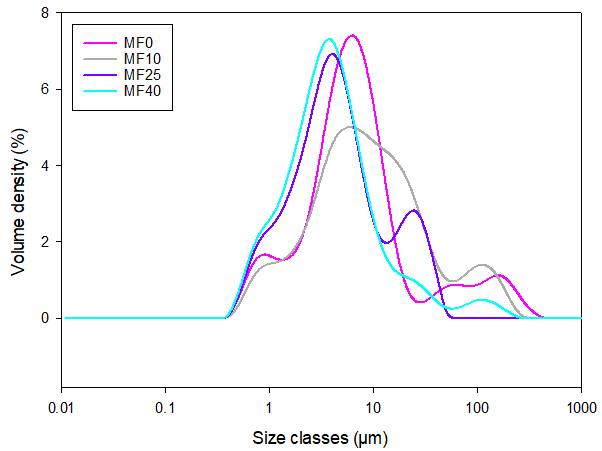
**
